# Partial Versus Complete Resuscitative Endovascular Balloon Occlusion of the Aorta in Exsanguinating Trauma Patients With Non-Compressible Torso Hemorrhage

**DOI:** 10.7759/cureus.8999

**Published:** 2020-07-04

**Authors:** Stacey E Heindl, Dwayne A Wiltshire, Ilmaben S Vahora, Nicholas Tsouklidis, Safeera Khan

**Affiliations:** 1 Medicine, California Institute of Behavioral Neurosciences and Psychology, Fairfield, USA; 2 Internal Medicine, California Institute of Behavioral Neurosciences and Psychology, Fairfield, USA; 3 Health Care Administration, University of Cincinnati Health, Cincinnati, USA; 4 Medicine, Atlantic University School of Medicine, Gros Islet, LCA

**Keywords:** reboa, hemorrhage, partial reboa, trauma resuscitation, trauma management, resuscitative endovascular balloon occlusion of the aorta, non-compressible torso hemorrhage, resuscitation, trauma

## Abstract

Hemorrhage is a major cause of death globally, yet our options to control the condition have remained limited. The standard intervention for patients suffering from a non-compressible torso hemorrhage (NCTH) typically involves resuscitative thoracotomy (RT) with aortic cross-clamping. Apart from being extraordinarily invasive, the survival rates for this procedure remain low. Over the years, research has surfaced that offers much promise regarding the use of resuscitative endovascular balloon occlusion of the aorta (REBOA) in exsanguinating patients. Although this type of procedure is not yet universally recognized as a gold standard, it holds some hope for the development of additional research regarding how we can make use of this advancement to improve survival in trauma patients. Complete REBOA (c-REBOA) has not gained wide acceptance due to the undeniable effects it has on normal physiology, metabolic effects, long-term complications, and mortality. Partial REBOA (p-REBOA) is not yet fully validated by research but could potentially be the answer to our problem. The critical question that we should address at this juncture is as follows: how can we improve the survival of patients with an NCTH in the least invasive way possible, while also reducing the feared complications associated with c-REBOA?

## Introduction and background

Injury remains the leading cause of death among people aged between 1-44 and accounts for five million deaths per year globally [1,2]. Of these fatalities, 30-40% are caused by hemorrhage, which makes it the second leading cause of death among the injured [3]. The statistics are staggering, yet the options to control a non-compressible torso hemorrhage (NCTH) in the prehospital phase are close to nonexistent and are considerably limited in the hospital as well, with a majority of the incidences resulting in aortic cross-clamping via resuscitative thoracotomy (RT) [4]. RT is an invasive process and is associated with low rates of patient survival [5-7]. Over the years, studies have emerged showing much promise regarding the use of resuscitative endovascular balloon occlusion of the aorta (REBOA) as a less invasive option compared to RT for stabilization in hemorrhage. Also, it has the unique ability to occlude different levels of the aorta depending on clinical needs; however, the procedure is still not universally used [8].

Purpose of REBOA

**Figure 1:**
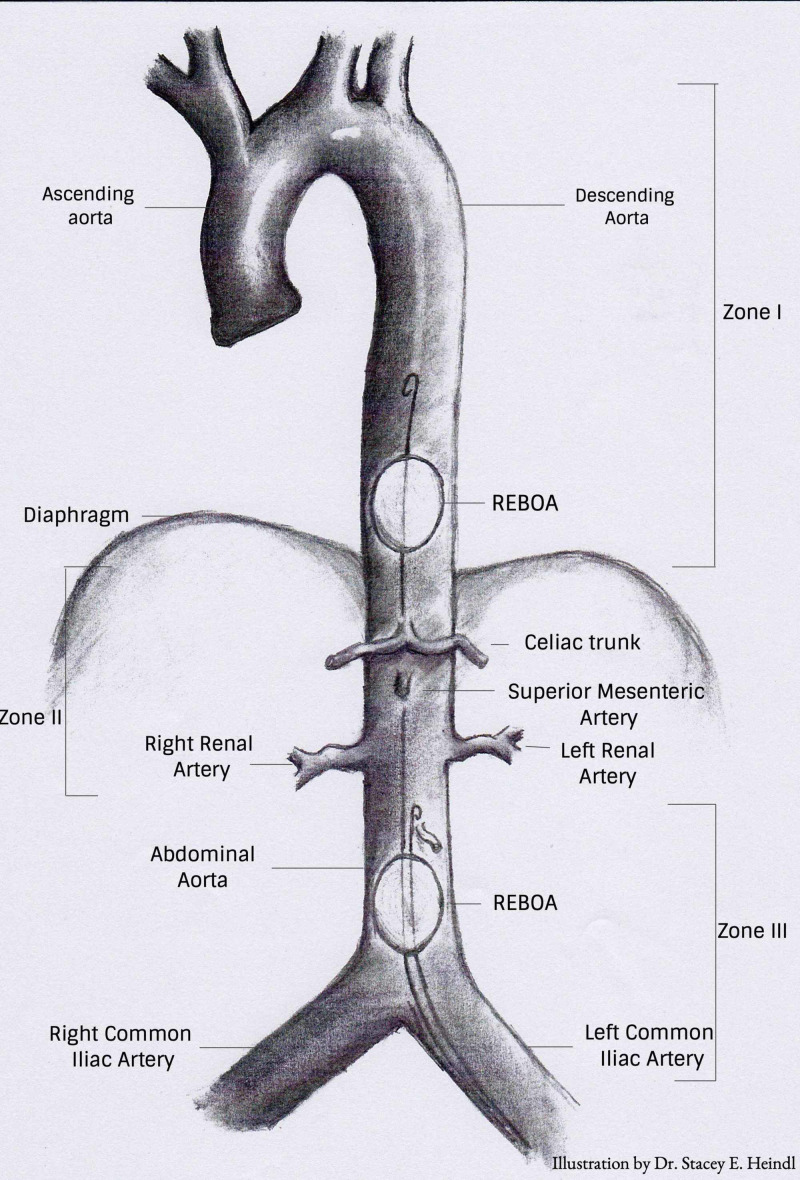
Zones of aortic occlusion Zone I extends from the left subclavian artery to the celiac artery. Zone II is from the celiac artery to the lowest renal artery. Zone III is from the lowest renal artery to the aortic bifurcation REBOA: resuscitative endovascular balloon occlusion of the aorta

REBOA involves the retrograde insertion of a 7-14 Fr catheter into the femoral artery. The catheter is then dilated with the preferred use of combined normal saline and X-ray contrast agent at either zone I, located above the diaphragm, for the control of intra-abdominal hemorrhage, or zone III at the aortoiliac bifurcation, to control pelvic hemorrhage (Figure 1) [4,5,8,9]. The purpose of this procedure is to bridge the gap from the onset of bleeding to surgical repair. REBOA allows for quick stabilization and immediate increase in systemic arterial blood pressure and cardiac output, thereby preventing cardiovascular collapse by sustaining adequate perfusion to the coronary and cerebral arteries [5,10-18]. Despite the prospects of such a simple device, there are still gaps in training in terms of ensuring proper catheter placement and accurate monitoring during deflation of the balloon; this combined with the various complications that can follow the procedure means that there is still much research to be done relating to the use of REBOA.

Associated complications

Complete REBOA (c-REBOA) has the potential to become a more acceptable alternative to a thoracotomy among physicians; however, it comes with various degrees of complications. The first significant obstacle will occur if the balloon gets inflated in the incorrect zone. Zone II occlusions are discouraged due to this being a perivisceral segment supplying the gastrointestinal (GI) tract, liver, and kidneys [5,8,9,19]. The duration of the occlusion is another major complication in c-REBOA. It has been shown that complete occlusion for 30-60 minutes can lead to a multitude of metabolic derangements [4,5]. Besides the increase of pancreatic, kidney, liver, and skeletal muscle enzymes, elevated lactate secondary to prolonged ischemia, the elevation of pro-inflammatory cytokines [interleukin-6 (IL-6), Interleukin-1β (IL-1β), and tumor necrosis factor-alpha (TNF-α)] and anti-inflammatory cytokines [interleukin 10 (IL-10)] are also predominantly observed after the occurrence of reperfusion [10,18,20,21]. Another serious complication seen in c-REBOA is the risk of profound distal ischemia to the lower extremities and spinal cord with complications as severe as amputation and paraplegia, respectively [4,5,22,23].

Partial REBOA

Partial REBOA (p-REBOA) is slowly gaining wide acceptance due yo its efficacy and reduced morbidity and mortality in exsanguinating patients. While c-REBOA offers many benefits, it also brings in many complications, which can be mitigated with the use of p-REBOA. p-REBOA is performed similarly to the c-REBOA except for the difference in balloon inflation [5,24]. It has been shown that the balloon should be inflated in correspondence to titrating the proximal blood pressure to approximately 80-90 mmHg while monitoring distal blood pressure to support distal perfusion [8,25]. This alteration not only allows for enough occlusion to promote blood supply proximally at a more physiologic level but also provides blood flow to the distal extremities, besides reducing the risk of compromised limbs and reperfusion injury. With this continuous perfusion, the overall metabolic derangements are significantly reduced [5,26]. This review article aims to identify knowledge gaps relating to the topic and to investigate further how p-REBOA compares to c-REBOA in decreasing mortality, metabolic and physiologic effects, and long-term complications.

## Review

Despite hemorrhage being a major cause of injury-related deaths, research is evolving rather slowly with regard to the options we have to manage this complex condition. Although there have been various discussions and studies about c-REBOA, there is limited research on p-REBOA and the role it could play not only in level I trauma centers but also in the prehospital phase. To emphasize the lack of research on this topic, our article search involving databases such as PubMed and Google Scholar yielded only 37 articles directly related to p-REBOA. We accepted all demographics, study types, year of publication, and also included animal studies. After careful consideration as to which articles would be used based on relation to the topics discussed in our research, we were left with a total of 12 articles; of those, nine had a primary focus on p-REBOA while three articles briefly discussed it. Only one of the 12 articles involved humans, while the remaining studies all utilized swine. The objective of our review article was to understand further how p-REBOA could offer a solution to many of the problems we are currently facing with the use of a c-REBOA in NCTH patients. We also addressed the limitations that currently prevent this seemingly “too good to be true” alternative from being used in everyday practice.

Physiologic effects

The purpose of using a REBOA is to control an NCTH until the source of bleeding can be identified and treated. It is of utmost importance to ensure the bleeding is well controlled, while also ensuring proper blood flow to vital organs. The common problem with a complete occlusion is that the lower limbs are compromised, while upper extremities receive a higher than average increase in mean arterial pressure (MAP) [26]. In two different studies with 15 swine, Russo et al. identified that a partial occlusion showed a more physiologic MAP and carotid blood flow when compared to a complete occlusion, which caused a supraphysiologic change proximal to the occlusion (Table 1) [26,27]. With the partial occlusion approach, there is some blood flow distal to the occlusion, which may help mitigate a multitude of complications. Forte et al. studied partial occlusion, with inflation of the balloon at 56.5% and distal flow rates of 0.7 L/min and 0.5 L/min in 25 swine. They concluded that this degree of occlusion was not only useful in hemorrhage control, but 0.5 L/min of flow distally had a 100% survival rate and also limited ischemic burden (Table 1) [28].

Based on the results of these studies, it would be easy to pose the question as to whether a p-REBOA can also allow for a more prolonged occlusion. Matsumura et al.'s study was one of the few to analyze p-REBOA procedures in humans. Based on the evidence from a multicentre registry in Japan, it involved a total of 142 adult participants, 78 of whom received a p-REBOA. The study showed that there is a better hemodynamic response (92% vs. 70%) and achievement of stability (78% vs. 51%) in partial vs. c-REBOA. It also demonstrated that partial occlusion could also sustain for nearly double the amount of time without a change in survival (58 minutes vs. 33 minutes in complete occlusion) (Table 1) [29].

The last pertinent area to be discussed in this section is how the change in blood flow can compromise multiple organs. With c-REBOA, the supraphysiologic change results in increased perfusion to proximal organs and elevation of cardiac afterload; this can have detrimental effects on the myocardium, resulting in not only the elevation of troponin but also diastolic dysfunction, followed by systolic dysfunction [30,31]. The sequela of this is pulmonary congestion, ultimately leading to respiratory failure [30]. p-REBOA prevents this supraphysiologic change, inevitably alleviating these ramifications. It is critical to mention the effects REBOA has distal to the occlusion, more specifically, on the GI tract and the long-term complications that can occur. In c-REBOA, the blood supply to the superior mesenteric artery (SMA) becomes compromised, which, in turn, can affect the small bowel. In several studies with c-REBOA, various histologic changes have been reported, such as necrosis and disruption of the duodenal mucosa, which are not found with p-REBOA [26,31]. One case report also identified a patient who underwent a c-REBOA for 40 minutes and had unfortunate complications from the procedure that led to ascending colonic ischemia with perforation, resulting in a colostomy [19].

**Table 1:** Description of selected studies on physiologic effects MAP: mean arterial pressure; p-REBOA: partial resuscitative endovascular balloon occlusion of the aorta; c-REBOA: complete resuscitative endovascular balloon occlusion of the aorta

Study	Year	Number of patients	Conclusion
Russo et al. [26]	2016	15 swine	p-REBOA provided a physiologic MAP and less ischemic changes
Russo et al. [27]	2016	15 swine	p-REBOA maintained a physiologic carotid blood flow and MAP compared to c-REBOA which produced supraphysiologic changes
Forte et al. [28]	2019	25 swine	p-REBOA provided effective hemorrhage control, limited ischemic burden, and a flow rate of 0.5 L/min provided 100% survival rate
Matsumura et al. [29]	2017	142 adult humans	78 patients received p-REBOA; there was better hemodynamic response, achievement of stability, and longer occlusion time with partial vs. full occlusion

p-REBOA holds much potential in managing hemorrhage while allowing for a proper physiologic state that supports the function of multiple organ systems overall. However, it is worth noting that this device may not be primarily used because of the degree of training required to utilize it, in addition to the limitations with partial balloon inflation that are not widely discussed. Some studies have reported issues with balloon migration and difficulties in maintaining blood pressure proximal and distal to the balloon, which can cause problems with hemorrhage control. In a zone I occlusion, as the pressure proximal to the balloon increases, it can be pushed to retrograde, resulting in the feared occlusion of zone II. There are also problems relating to the natural movement of the vessel walls and lack of friction. Since the balloon does not actively expand and contract with vessel wall movement, there is no consistency in the precise amount of blood flow around the balloon, making hemorrhage control and adequate perfusion proximally and distally quite tricky. To conclude, there are still advancements to be achieved to enhance the functionality of the device, with better response to physiologic changes.

Metabolic effects

Metabolic derangements are a common problem among trauma patients, primarily due to hemorrhage, resulting in hypovolemia and decreased perfusion. With the use of REBOA, these abnormalities become exponentially heightened. The initial stage of complete occlusion results in the lack of perfusion to the lower extremities and multiple organs, causing the cells to switch from aerobic to anaerobic glycolysis to yield energy. The process of anaerobic glycolysis results in elevations of lactate. Normal serum lactate levels are 0.5-1 mmol/L, and once this level exceeds >4 mmol/L, it is considered lactic acidosis [32]. Kauvar et al. studied lactate levels in 21 swine and concluded that lactate was higher and remained elevated even after balloon deflation in c-REBOA when compared to p-REBOA (Table 2) [33]. Forte et al. also analyzed lactate levels with the use of REBOA with regard to blood flow to the distal extremities. In this study, 25 swine were used; a flow rate of 0.7 L/min resulted in a lactate level of 9.6 mmol/L, a flow of 0.5 L/min resulted in a level of 12.6 mmol/L, and a flow of 0.3 L/min caused the lactate to increase to 13.3 mmol/L. From this data, we can conclude that lactate levels elevate with decreased perfusion (Table 2) [28]. This level of elevation of lactate in correspondence with prolonged lactate clearance is of particular importance when looking at the overall mortality. Heinonen et al. studied elevated lactate levels in blunt and penetrating trauma patients, concluding that prolonged elevation correlates with increased mortality in trauma patients. More specifically, in blunt trauma patients or patients with NCTH, the survival rate is around 38% [34]. Hence, with the utilization of a p-REBOA, we can keep lactate levels lower and have normalization of lactate after balloon deflation, which could play an important role in reducing overall mortality.

As mentioned before, there is the risk of an ischemic burden that occurs with REBOA. When discussing ischemic damage, it is crucial to understand how the process works so that one can better understand the consequences that follow. When perfusion to tissues is not sufficient, the cells become deprived of oxygen. This lack of oxygen will impair oxidative phosphorylation, resulting in a decrease of adenosine triphosphate (ATP). Impaired capability to produce ATP will directly affect the Na⁺/K⁺ ATPase pump, an essential component in maintaining a balance between water and ions in the cell. This disturbance in the cellular balance causes Na⁺ retention, with the addition of water further causing cellular swelling. If the injury to the tissue is prolonged, the cell injury becomes irreversible. The byproduct of irreversible cell injury is damage to the plasma membrane, proceeded by the release of cytosolic enzymes into the serum, in addition to the elevation of Ca²⁺ within the cell, secondary to Ca²⁺pump failure.

Since c-REBOA prevents blood supply to the SMA, the organs supplied by this artery are compromised and can undergo irreversible cell injury. The repercussion of this is duodenal necrosis, as previously discussed, in addition to the elevation of pancreatic enzymes, as described by Sadeghi et al. [10]. Other tissues and organs that have been reportedly affected are skeletal muscle, kidneys, and the liver, which was reported in Sadeghi et al.'s study with 24 swine (Table 2) [10]. These abnormalities were either not seen or seen only to a lesser degree in a partial occlusion compared to complete occlusion. Sadeghi conducted another study in 2018 with 18 swine, which demonstrated that there are less metabolic and inflammatory sequelae for partial occlusion in comparison to complete occlusion (Table 2) [31].

As we now know, there are many problems that can arise with occlusion of blood flow; however, there are also substantial consequences after the reperfusion of the oxygen-deprived tissue. The return of blood flow to the already ischemic tissue not only brings oxygenation but also results in the production of free radicals. Free radicals will then intensify the tissue damage, producing an exponential increase in the enzymatic release from the cells. We must also anticipate the mediation of inflammation that will occur. Morrison et al. studied c-REBOA in 20 swine and concluded that there is a significant increase in the pro-inflammatory cytokine IL-6 and cases of acute respiratory distress syndrome (ARDS) after the removal of complete occlusion at 60 minutes and 90 minutes (Table 2) [18]. This point is critical when comparing partial vs. complete occlusion of the aorta, as partial occlusion can decrease the degree of inflammation that occurs and also the irreversible damage to the cells, evidently preventing reperfusion injury.

**Table 2:** Description of selected studies on metabolic effects p-REBOA: partial resuscitative endovascular balloon occlusion of the aorta; c-REBOA: complete resuscitative endovascular balloon occlusion of the aorta; ARDS: acute respiratory distress syndrome; IL-6: interleukin-6

Study	Year	Number of patients	Conclusion
Kauvar et al. [33]	2019	21 swine	Higher lactate levels in c-REBOA that remained elevated in comparison to p-REBOA
Forte et al. [28]	2019	25 swine	Lactate levels increased with decreased perfusion. Comparison of flow rate with lactate levels are as follows: 0.7 L/min = 9.6 mmol/L; 0.5 L/min = 12.6 mmol/L, 0.3 L/min = 13.3 mmol/L
Sadeghi et al. [10]	2020	24 swine	Severe systemic metabolic disturbances, organ damage and inflammatory activation seen within 30 minutes of c-REBOA
Sadeghi et al. [31]	2018	18 swine	Elevated lactate and troponin in c-REBOA when compared to the control group and p-REBOA. Cytokine response and histologic changes more pronounced in c-REBOA
Morrison et al. [18]	2014	20 swine	Significant increase in IL-6 in the 60- and 90-minute complete occlusion groups. ARDS seen in all subject groups (30-, 60-, and 90-minute groups)

Long-term complications and mortality

When analyzing the overall outcome of REBOA procedures, it is essential to review the long-term complications that can occur in addition to the overall mortality of patients receiving the procedure. In other words, we need to understand not only the efficacy but also the safety of the procedure. When comparing c-REBOA with p-REBOA, it has been concluded in three different studies that p-REBOA does show a decrease in mortality. In the first study, Kauvar et al. utilized a total of 21 swine, five in the control group, eight with c-REBOA, and eight with p-REBOA. Groups with the intervention received 60-minute balloon inflation. At the end of the study, the control group had a mortality rate of 100%, the c-REBOA group had 62.5%, and the p-REBOA group had 12.5%. (Table 3) [33]. Therefore, not only does p-REBOA have a lower mortality rate, but it is also one of significance. Forte et al. also conducted a similar study in 20 swine, where 10 received a complete occlusion and 10 received a partial occlusion, with the results showing 20% survival in the complete occlusion group and 70% survival in the partial occlusion group, again proving that there is a significant improvement in survival among the partial occlusion group (Table 3) [35]. Lastly, Russo et al. showed that p-REBOA increased the overall survival by 3.5-fold compared to no intervention. Significantly, Russo also mentions how hemostasis was challenging to achieve when compared to complete occlusion, as trying to titrate and maintain balloon inflation to a certain degree is of great difficulty [30]. There is some bias associated with this study as it only represents the challenges faced with one swine in a prehospital setting.

Long-term complications are of great importance; as we know, there can be a significant risk when completely occluding the blood supply to the distal extremities. As discussed, there can be ischemia to the spinal cord and femoral arteries resulting in paraplegia and possible amputation, respectively. Saito et al. conducted a study involving 24 patients, all of whom received a c-REBOA. Of the 24 patients, three (12.5%) patients had severe complications from the procedure. These three patients developed acute kidney injury. Of the three patients, two had critical lower limb ischemia, both of which resulted in lower limb amputation (Table 3) [36]. With the use of p-REBOA, we can prevent this distal ischemia, potentially preventing long-term complications such as amputation in these patients.

**Table 3:** Description of selected studies on long-term complications and mortality p-REBOA: partial resuscitative endovascular balloon occlusion of the aorta; c-REBOA: complete resuscitative endovascular balloon occlusion of the aorta

Study	Year	Number of patients	Conclusion
Kauvar et al. [33]	2019	21 swine	60-minute c-REBOA had a mortality rate of 62.5%, p-REBOA had a mortality rate of 12.5%. P-REBOA for one hour improved survival
Forte et al. [35]	2020	20 swine	2/10 swine survived c-REBOA while 7/10 swine survived p-REBOA
Saito et al. [36]	2015	24 patients	c-REBOA resulted in three (12.5%) patients having complications. All three patients had kidney injuries; two of the three patients had complications of lower limb ischemia resulting in amputation

Limitations

The major limitation of this paper was that there were very few studies conducted using p-REBOA that we could base our review on. Also, there may be some degree of bias, as the majority of studies have not been conducted in humans but in swine models. Although most swine had undergone some degree of induced trauma to establish the efficacy of REBOA, most procedures were done in a very controlled setting. To obtain a better idea of the effectiveness and safety of p-REBOA, we need to have more studies conducted on trauma patients by medical professionals who are well-versed in the REBOA procedure. This will help us to establish whether or not the REBOA procedure can hold its own in a high-stress setting involving multiple variables.

## Conclusions

This review article aimed to analyze and discuss how the p-REBOA procedure compared to c-REBOA in providing more favorable physiologic outcomes with lower metabolic alterations, mortality, and long-term complications. It was shown that c-REBOA has less desirable physiologic effects when compared to p-REBOA; also, c-REBOA can lead to a more pronounced elevation in lactate and enzyme release. Long-term complications and mortality are greater with complete occlusion when compared to the partial one. Recommendations for the future include more education and training for medical professionals on the benefits of this procedure. Additionally, more research also needs to be done on p-REBOA in humans to truly understand the challenges this procedure may present and how we can overcome them. The device could also be modified to allow for a partial occlusion to provide more consistent hemorrhage control with the added accommodation of normal vessel physiology. We believe this article contributes to the search for new innovative solutions to control NCTH in exsanguinating patients. It also provides a strong foundation for the potential development of a more advanced device, which could benefit the vast number of people who are affected every year by hemorrhage.
